# Mr. Blake on the Hooping Cough

**Published:** 1812-02

**Authors:** Andrew Blake

**Affiliations:** Member of the Royal College of Surgeons, London. Framlingham


*To the Editors of the Medical and Physical Journal.*

GENTLEMEN,

THE frequency of Hooping Cough, connected with its obstinacy in resisting the usual endeavors made for its cure, have induced me to request that, through the medium of your extensive miscellany, you will give publicity to the following medicine, which, I believe, though recommended (or something similar to it) in some foreign work, is not sufficiently known to bring it into that general practice it appears to deserve.

℞. Vin. Antimonii ℥j.

Ext. Hyosciami ℈ij. Misce.

The dose of course to be regulated by the age of the child. The cases in which I have had an opportunity of witnessing its utility, were effectually relieved by very small doses of three or four drops four times a day, gradually increasing the quantity till a slight degree of nausea was complained of. It has had the wished-for effect after the usual means were unsuccessfully resorted to. I am, Gentlemen,

Your most obedient Servant,
ANDREW BLAKE,
Member of the Royal College of Surgeons, London.

*Framlingham, Sept.* 1811.

